# General practitioners’ perceptions of public reporting of institution and individual medicine prescribing data

**DOI:** 10.1186/s12913-016-1893-5

**Published:** 2016-11-09

**Authors:** Xin Du, Xinping Zhang, Yuqing Tang

**Affiliations:** School of Medicine and Health Management, Tongji Medical College, Huazhong University of Science and Technology, No.13. Hangkong Road, Wuhan, 430030 Hubei province People’s Republic of China

**Keywords:** Public reporting, Perception, Medicine prescribing data, General practitioner

## Abstract

**Background:**

Public reporting of institution- and individual-level performance data has recently become a popular topic in the health care field. This study (1) evaluated the perceptions of general practitioners on the public reporting of institutional and individual medicine prescribing data in primary care institutions, and (2) compared the difference among the perceptions of general practitioners on the dimensions of necessity, methodological rigor, and impact of public reporting medicine prescribing data.

**Methods:**

We conducted a survey in 10 primary care institutions in Q city, Hubei province. General practitioners who hold prescribing license were eligible for this study; we surveyed all eligible general practitioners in July 2014. The survey instrument was developed based on previous studies and expert opinions. Paired *t*-test or nonparametric test was used to evaluate the difference in perceptions between the institutional and individual medicine prescribing data reporting. An analysis of variance test was used to analyze the score differences among the three dimensions (i.e., necessity, methodological rigor, and impact).

**Results:**

A total of 154 general practitioners were surveyed in this study. No significant difference in the perceptions of general practitioners was observed between the institution- and individual-level medicine prescribing data reporting (*p* > 0.05). General practitioners have significantly different perceptions on the three dimensions of the institution- and individual-level data reporting (*p* < 0.05). Methodological rigor obtained the lowest score. Regarding the strategies to facilitate the medicine prescribing data reporting, over 80 % of general practitioners selected the items that disclose process measures and not outcome measures, as well as educate patients on data interpretation.

**Conclusion:**

The perceptions of general practitioners between institution- and individual-level data reporting have no significant difference. General practitioners place their utmost concern on public reporting on the methodological rigor. Processing measures and patient education to improve the efficiency of public reporting require substantial attention.

**Electronic supplementary material:**

The online version of this article (doi:10.1186/s12913-016-1893-5) contains supplementary material, which is available to authorized users.

## Background

In recent decades, public reporting has been used as a vehicle to improve the performance at the institutional and individual levels [1]. Hospital-level public reporting is currently familiar to most clinicians, whereas physician-level reporting is rapidly progressing and becoming increasingly prevalent [2]. In the US, the outcomes data for individual cardiac surgeons have been publicly available in the states of New York, Pennsylvania, and New Jersey since 1991, 1992, and 1994, respectively [3]. Thereafter, an independent newsroom in the US published the nationwide complication rates of nearly 17,000 surgeons in 2005 [1]. In 2005, the Society for Cardiothoracic Surgery (SCTS) in Great Britain and Ireland published publicly accessible performance data of individual hospitals and surgeons [4]. In 2013, England became the first country to mandate the release of the names of surgeons and outcome data associated with surgeons across nine surgical specialties [1].

The public reporting of health care performance data offers several potential benefits. First, public reporting enables patients to identify the best physicians and hospitals, as well as to make informed choices [5]. Second, public reporting will increase competition within the health care system and offer physicians and hospitals incentives to improve quality [2, 6]. Third, public reporting provides policymakers and third-party payers with knowledge for informed decisions on payment, including rewarding high or penalizing low performers [7, 8]. Public reporting does, however, have several unintended consequences. For example, providers may become risk averse, clinical priorities may become distorted, and staff morale may be reduced [9]. In addition, the inadequate risk adjustment, lack of valid quality metrics and validated data are cited by critics [6].

Although public reporting is being extensively studied, the perceptions of physicians on the public reporting of hospital- and physician-level performance data are limited. Ross et al. conducted qualitative interviews to explore the perceptions of physicians on public reporting, and indicated that the interviewees expressed skepticism regarding public reporting because of concerns on the ability of patients to comprehend the performance data. However, Ross et al. were only focused on the perceptions of physicians on the hospital level performance data reporting [10]. Sherman et al. evaluated the perceptions of surgeons on the public reporting of the hospital- and individual-level surgical qualities. The investigated surgeons expressed concerns regarding public reporting, particularly the reporting of individual performance, and were more in favor of public reporting of hospital level outcomes data [6]. Nevertheless, Sherman’s study focused on the perceptions of surgeons, and perceptions may not be generalizable to other medical workers, such as the general practitioners (GPs) in primary care institutions. GPs play a gatekeeping role in the health care system [11]. Therefore, gaining an improved understanding of the perceptions of GPs on the public reporting of the hospital- and individual-level performance data may facilitate the acceptance of public reporting, improve performance, and enrich the evidence of public reporting research.

This study intends to answer the following questions: (1) Do GPs have different perceptions on the public reporting of the institution- and individual-level medicine prescribing data in primary care institutions? (2) What is the difference among the perceptions of GPs on the necessity, methodological rigor, and impact of medicine prescribing data reporting?

## Methods

### Setting and participants

This study was conducted in Q city, Hubei Province, and involved 10 primary care institutions. Q city is located in central Hubei, with a land area of 2004 km^2^ and a population of 0.95 million. The annual GDP of Q city reached 49.3 billion (RMB yuan) in 2013, just above the average level of all cities in Hubei. The majority of inhabitants in Q city are of Han ethnicity, with about 8000 from Hui, Tujia, and other ethnic groups. The population serviced by the 10 primary care institutions was about 383, 000; outpatient visits was about 49,108; and inpatient was about 1, 482 per year. The majority of patients (about 60 %) of the 10 primary care institutions were covered by New Cooperative Medical Scheme (NCMS); about 20 % patients were covered by other medical insurances (basic medical insurance for urban employees, basic medical insurance for urban residents); and the rest 20 % patients paid at their own expense. For the NCMS, the reimbursement rate is about 85 % for inpatients and about 50 % for outpatient in the Q city.

We selected Q city because the institutional and individual medicine prescribing data have been released to public in November 2013 to promote the rational use of medicine. The public reporting had already last for 9 months when we conducted the investigation. Besides, the health authority of Q city was very supportive of this project and it guaranteed the investigation conducted smoothly. Three prescribing indicators using the outpatient prescription data were calculated at the institution- and individual-levels: percentage of prescriptions requiring antibiotics; percentage of prescriptions requiring injections; and average drug expenditure per prescription. These indicators were ranked in order at the institutional level within 10 institutions and the individual prescriber level within an institution on a monthly basis. The ranking results were displayed in a public place (entrance hall).

GPs with prescribing license from the 10 primary care institutions were eligible for this study. We calculated the sample size with the follow formula:

$$ \mathrm{n}=\frac{{\mathrm{Z}}_{1-\alpha /2}^2\left(1-P\right)}{\upvarepsilon^2P} $$

where, *Z*_1 − *α*/2_ is the value obtained from the standard normal distribution table for 100 (1-α)% confidence level. Its value for 95 % and 99 % confidence level is 1.96 and 2.58 respectively, *P* is the anticipated proportion of the condition in the study population and ε is the relative precision [12].

According previous study, about 79 % of the doctors are aware of public reporting [13]. The minimum sample size required for this study for relative precision of 10 % and confidence level of 95 % was 103.

All of the 154 eligible GPs of the 10 primary care institutions would be surveyed on considering of response rate. The investigation was conducted in July 2014.

This study was approved by the Ethics Committee of Tongji Medical College, Huazhong University of Science and Technology (NO: IORG 0003571). An introductory letter was attached to the questionnaire to explain the objectives of this study, and verbally informed consent was obtained from the survey participants. Permission was also granted by the local health authority.

### Instrument

The items were developed based on Sherman’s survey instrument and expert opinions; these items were modified for applicability to the research site and target population [6].

The structured instrument included 3 dimensions and 33 items (i.e., 16 pairs of questions on the perceptions of institution- and individual-level data reporting, as well as a question on the strategies on facilitating public reporting). Five-point Likert scale response options were used in the instrument; the higher the score, the more positive response to public reporting.

The first dimension was perceptions on the necessity of public reporting, including whether public reporting is necessary, and whether third-party reporting is necessary (1 = not at all necessary, 2 = slightly necessary, 3 = somewhat necessary, 4 = very necessary, 5 = extremely necessary).

The second dimension covered the perceptions on the methodological rigor of public reporting, that is, whether GPs are concerned with the following issues: patients cannot interpret or recognize data complexity, the public reporting data are unreliable, a lack of appropriate medicine prescribing metrics, have no adequate risk adjustment, have no adequate amount of prescriptions for statistical comparison, a lake of transparency of methodology (1 = extremely concerned, 2 = moderately concerned, 3 = somewhat concerned, 4 = slightly concerned, 5 = not at all concerned).

The third dimension was perceptions on the impact of public reporting, covering positive impact perceptions and negative impact perceptions. The positive impact perceptions of public reporting include: improving prescription quality, learning more knowledge on rational prescription of medicines, and providing a sense of achievement to GPs (1 = strongly disagree, 2 = disagree, 3 = neither agree nor disagree, 4 = agree, 5 = strongly agree). The negative impact perceptions of public reporting include: causing risk averse, damaging quality, penalizing low performers, losing volumes of patients, and increasing medical disputes (1 = strongly agree, 2 = agree, 3 = neither agree nor disagree, 4 = disagree, 5 = strongly disagree).

The last question of the instrument is about the promotion strategy of public reporting, and it’s a multiple choices. Items mainly based on the follow-up interview thematic analysis outcomes conducted by Sherman, including internal review before public reporting, third-party reporting, educating patients on data interpretation, presenting simplified data to patients, more comprehensive risk stratification and case-mix adjustment, and focusing on process measures and not outcome measures. An additional file shows the details of the instrument (see Additional file 1).

### Statistical analysis

The mean Likert score with standard deviation (SD) and frequency of positive responses were calculated for each item at the institution- and individual-levels. One-sample Kolmogorov-Smirnov test was used to determine if a normal distribution of perceptions score was, the null hypothesis is that “sample distribution is normal”, and if the test is significant, the distribution is non-normal. For the normal distribution perceptions score, the difference would be compared using paired t-tests. Otherwise, nonparametric test should be used. An analysis of variance (ANOVA) test was used to analyze the differences of Likert score among the three dimensions (i.e., necessity, methodological rigor, and impact of public reporting). If the ANOVA test showed a significant difference in means between the three dimensions, then the Bonferroni correction method will be employed to determine how they differ. A *p*-value of < 0.05 was considered significant for all comparisons except for the mean pair-wise analysis with Bonferroni correction. For Bonferroni’s correction method, the adjusted *p*-level was 0.0167, that is, the 0.05 was divided by 3 (the number of pair-wise comparisons in this study was 3). All statistical analyses were performed using SPSS17.0 (SPSS Inc., Chicago, IL).

## Results

### Characteristics of the participating GPs

A total of 154 questionnaires were distributed and all of them were collected, the response rate was 100 %. Table 1 shows the characteristics of these GPs. The majority of GPs were males (61.44 %), and those between 31 and 60 years old accounted for over 80 %. GPs in Q city possessed low-level education, that is, only 18.18 % of the total GPs had completed a bachelor’s (or higher) degree. In terms of job title, the majority of GPs (64.19 %) possess a primary title or lower. GPs represented all units, including internal, surgery, traditional Chinese medicine, gynecology and obstetrics, stomatology, psychiatry, and other units. The income of most GPs (81.17 %) was less than 2500 RMB yuan per month (USD 375, the exchange rate was US $1 = RMB 6.67 in October 2016), but their working time was considerably long, and over 70 % GPs worked for more than 40 h a week.

**Table 1:** Demographic characteristics of the respondents

Demographic characteristics		Frequency	Percent (%)
Gender	Male	94	61.44
	Female	59	38.56
Age	≤30	23	15.23
	31–40	69	45.7
	41–50	37	24.5
	51–60	18	11.92
	>60	4	2.65
Education	Senior middle school or lower	40	25.97
	Associate degree	86	55.84
	University degree or higher	28	18.18
Title	Not certified	8	5.41
	Certified physician	34	22.97
	Resident doctor	53	35.81
	Attending doctor	44	29.73
	Associate senior doctor	9	6.08
Unit	Internal	64	41.56
	Surgery	23	14.94
	Traditional Chinese medicine	10	6.49
	Gynecology and Obstetrics	26	16.88
	Stomatology	14	9.09
	Psychiatry	5	3.25
	Others	12	7.79
Monthly income (RMB yuan)	<2000	82	53.25
	2000–2500	43	27.92
	>2500	29	18.83
Weekly working time (hour)	≤40	43	28.1
	>40	110	71.9

### Perceptions of GPs on the institution- and individual-level medicine prescribing data reporting

In general, the positive response of GPs to the necessity of institution-level reporting was higher than that in individual-level reporting (i.e., 82.14 % vs. 76.95 %); however, no significant difference (*p* = 0.229) was observed. For the methodological rigor dimension, the positive response to institution-level reporting was higher than that in individual-level reporting (i.e., 68.18 % vs. 65.91 %), that is, GPs have more concerns about individual- level reporting. However, the difference has no statistical significance (*p* = 0.387). Regarding impact dimension, GPs have higher positive response to institution-level reporting than that in individual-level reporting (i.e., 83.12 % vs 81.49 %), although no statistical difference (*p* = 0.881) was observed. Table 2 shows the responses to each survey item regarding the public reporting of institution- and individual-level medicine prescribing data.

**Table 2:** Perceptions of GPs on the public reporting of institution- and individual-level medicine prescribing data

Survey Items	Institution-level		Individual-level		T T = (Mean1–Mean2)	*p*
	Mean 1 (SD)	Positive response (%)	Mean 2 (SD)	Positive response (%)		
Necessity	**3.94 (1.024)**	**82.14**	**3.81 (1.039)**	**76.95**	**0.13**	**0.229**
Public reporting is necessary	3.89 (1.152)	78.57	3.69 (1.157)	71.75	0.19	0.096
Third-party reporting is necessary	3.98 (1.057)	81.49	3.92 (1.072)	79.22	0.06	0.566
Methodological rigor	**3.35 (0.774)**	**68.18**	**3.34 (0.783)**	**65.91**	**0.01**	0.387*
Patients cannot interpret or recognize data complexity	3.10 (1.127)	49.03	2.99 (1.146)	46.43	0.11	0.428
Public reporting data is unreliable	3.72 (1.103)	73.53	3.73 (1.098)	73.70	−0.01	0.959
Lack of appropriately prescribed medicine metrics	3.64 (1.047)	69.48	3.66 (1.050)	70.45	−0.02	0.846
No adequate risk adjustment	2.86 (1.172)	41.50	2.86 (1.237)	41.88	−0.01	0.959
No adequate amount of prescriptions for statistical comparison	3.23 (1.174)	56.49	3.23 (1.209)	55.84	−0.01	0.977
Lack of transparency of methodology	3.58 (1.083)	69.81	3.56 (1.078)	69.16	0.02	0.856
Impact	**3.66 (0.622)**	**83.12**	**3.66 (0.636)**	**81.49**	**0**	0.881*
Improved prescription quality	3.82 (1.032)	77.92	3.86 (1.032)	78.90	−0.04	0.716
GPs learn more knowledge on the rational prescription of medicines	4.05 (1.008)	84.09	4.12 (0.952)	86.27	−0.07	0.636
Provide a sense of achievement to GPs	3.31 (1.100)	56.82	3.31 (1.093)	57.47	0.01	0.994
Refuse high-risk patients	3.5 (1.04)	68.63	3.5 (1.031)	68.51	0	0.995
Improve numbers and not quality	3.49 (1.074)	68.83	3.5 (1.080)	68.83	−0.01	0.92
Penalize low performing GPs	3.76 (1.010)	78.90	3.74 (1.015)	77.92	0.02	0.832
Loss of volume of patients	3.75 (0.873)	78.57	3.70 (0.951)	75.97	0.05	0.800
Increase medical disputes	3.59 (1.088)	70.13	3.57 (1.090)	69.16	0.02	0.854

Percentage of positive response = (1/2 number of GPs who obtained a score of 3 + all numbers of GPs who obtained a score of 4 + all numbers of GPs who obtained score of 5)/ total number of GPs; * represents the *p* value calculated with paired t-tests; otherwise, calculated with nonparametric test; bold text represents the results of dimension

### Perceptions of GPs on the necessity, methodological rigor, and impact of public reporting

The perceptions of GPs on the three dimensions (i.e., necessity, methodological rigor, and impact) have a significant difference, regardless whether it is on the institution- or individual-level data reporting (F = 19.18, *p* < 0.001 and F = 12.54, *p* < 0.001, respectively). Further Bonferroni correction method test revealed that every two dimensions have statistical significance except that the necessity and impact dimension of individual- level data reporting (*p* = 0.138). Table 3 shows the specific results of ANOVA and Bonferroni correction method test.

**Table 3:** Perceptions of GPs on the three dimensions of institution- and individual-level medicine prescribing data reporting

Dimension	Institution-level				Individual-level			
	Score range	Mean dimension score (SD)	F	*p*	Score range	Mean dimension score (SD)	F	*p*
Necessity	1–5	3.94 (1.02)	19.18	<0.001	1–5	3.81 (1.04)	12.54	<0.001
Methodological rigor	1–5	3.35 (0.77)			1–5	3.34 (0.78)		
Impact	1–5	3.66 (0.62)			1–5	3.66 (0.64)		
^a^Bonferroni correction			Unadjusted *p*	Adjusted *p*			Unadjusted *p*	Adjusted *p*
		1 vs. 2	<0.001	<0.001		1 vs. 2	<0.001	<0.001
		1 vs. 3	0.011	0.004		1 vs. 3	0.414	0.138
		2 vs. 3	0.003	0.001		2 vs. 3	0.002	<0.001

dimension score = summed score of all items of the dimension / the number of items of the dimension; ^a^1 represents “necessity” dimension, 2 represents “methodological rigor” dimension, 3 represents “impact” dimension

### Strategies to facilitate the public reporting of medicine prescribing data

Table 4 shows that the top three strategies that facilitate public reporting of medicine prescribing data were focused on process measures and not outcome measures (85.71 %), educating patients on data interpretation (82.47 %), and more comprehensive risk stratification and case-mix adjustment (76.62 %). GPs believed that the third-party reporting and first internal review before public reporting were effective strategies to facilitate public reporting acceptance. A total of 74.03 and 68.83 % of GPs selected the two items, respectively. Only 44.81 % of the surveyed GPs considered that presenting simplified data to patients was an effective strategy to facilitate the public reporting.

**Table 4:** Priority strategies to facilitate the public reporting of medicine prescribing data

Survey Items	*N*	Frequency	Percent (%)
Focus on process measures and not outcome measures	154	132	85.71
Educate patients on data interpretation	154	127	82.47
More comprehensive risk stratification and case-mix adjustment	154	118	76.62
Third-party reporting	154	114	74.03
Internal review before public reporting	154	106	68.83
Present simplified data to patients	154	69	44.81

Survey items are ranked based on frequency from highest to lowest

## Discussion

This study determined that the difference of perceptions of GPs between public reporting of the institution- and individual-level data lacked statistical significance. However, GPs expressed highest concerns regarding the methodological rigor dimension of public reporting. Thus, strategies should be considered to facilitate the public reporting of medicine prescribing data, such as disclosing process measures and educating patients on data interpretation.

First, the lack of significant difference on the perceptions of GPs between the institution- and individual-level data reporting should be explained. Public reporting may result in negative impacts on market share, contracting arrangements, or reputation of health care providers [14, 15]. Generally speaking, for self-preservation, the positive response of clinicians to individual-level data reporting may be generally lower than that in institution-level data reporting. However, our results did not support this hypothesis. The current transparency policy and medical environment of China may explain this phenomenon. On the one hand, provider incentives played an important role in public reporting [16], but China’s transparency policy lacks explicit incentives. Regardless whether GPs have high or low performance, they both obtained limited penalty or reward. Therefore, GPs did not demonstrate evident reluctance and concerns on the public reporting of their own individual-level medicine prescribing data in our study. On the other hand, education level and socioeconomic status have been demonstrated to affect the searching habits of patients, and quality information was used frequently by those with high education levels [17]. Patients in Q city with low levels of education may use limited information to select the providers [18]. Therefore, GPs’ perceptions of individual-level reporting were no more negative than institution-level reporting because they have no selection pressure coming from patients. Note that the results of Sherman’s study indicated that most surgeons supported the public reporting of quality metrics at the hospital level but opposed individual reporting [6]. Therefore, the studies on the perceptions of clinicians on the institution- and individual-level data reporting showed mixed results and required further research.

Second, compared with the necessity and impact of public reporting, the methodological rigor was the dimension where GPs placed their utmost concern. This result was consistent with those of previous studies. Barr et al. conducted an interview to explore the view of physicians on public reporting and determined that physicians perceived that public reporting lacked methodological rigor [19]. Rechel et al. mapped the current approaches of public reporting in 11 high-income countries and learned that the accuracy and reliability of performance data were a main concern of public reporting [20]. Burns et al. suggested that if public reporting was desirable, the reporting data must be valid, and the main validity concerns included sample size; model performance for risk adjustment; and quality of the data [1]. Necessity and impact have higher scores than methodological rigor, thereby indicating that GPs viewed public reporting as necessary and has significant influence on prescribing practice. This result was similar to those of other studies. Public reporting of provider quality information was viewed as a necessary building block in the pursuit of value-based health care [21]. Survey data from physicians indicated that public reporting significantly influences performance improvement [22]. Recent data from the Wisconsin Collaborative for Healthcare Quality showed that public reporting has an impact on quality improvement [23, 24].

Finally, for the strategies to facilitate public reporting acceptance, over 80 % of GPs involved deemed that public reporting should focus on process measures and not outcome measures, as well as educate patients on data interpretation. The Agency for Healthcare Research and Quality (AHRO) showed that process measures have been used for public reporting and have the following advantages over the outcome measures: (1) process measures may be easier and less costly to measure, (2) process measures can be advantageous when outcomes of interest are rare or sample sizes are small, (3) and well-specified process measures do not need case-mix or risk adjustment [25]. The Process-Based Quality Improvement Manual stressed that process quality measures expand the domains of quality measurement and were important to promote health care performance [26]. Similar to emphasizing the importance of process measures, several studies stressed the significance of patient education during public reporting. Yang indicated that consumer education was required to maximize the impact of public reporting [27]. Stephanie et al. suggested that education was required to help assist patients interpret quality data, and an improved public education on quality data was required to increase the use of reporting data in the future [28].

The current study has a few limitations. First, as the study was conducted in primary care institutions, the conclusions drawn from this research must be carefully generalized to other types of healthcare institutions. Second, this study was undertaken in just one city. Since socio-economic and cultural may have an effect of GPs public reporting perceptions, the GPs of our study may not be representative of all GPs that with different economic and cultural types. We may expand the sample size in the future when other medical institutions public reporting institution- and individual-level medicine use information.

## Conclusion

This study determined no significant difference in the GPs’ perceptions between institution- and individual-level data reporting. Meanwhile, the utmost concern of GPs lies on the methodological rigor of public reporting. Extensive focus should be placed on the disclosure of process measures and not outcome measures. Furthermore, patients should be educated on data interpretation to improve the efficiency of public reporting.

## Additional file

Additional file 1: Appendix A: Questionnaire of General Practitioners’ Perceptions of Public Reporting. (DOCX 19 kb)

## Abbreviations

AHRO: Agency for Healthcare Research and Quality
ANOVA: Analysis of variance
GP: General practitioners
NCMS: New Cooperative Medical Scheme
SCTS: Society for Cardiothoracic Surgery
SD: Standard deviation

## References

[1] Burns EM, Pettengell C, Athanasiou T, Darzi A (2016). Understanding The Strengths And Weaknesses Of Public Reporting Of Surgeon-Specific Outcome Data. Health Aff.

[2] Dehmer GJ, Jennings J, Madden RA, Malenka DJ, Masoudi FA, McKay CR (2016). The National Cardiovascular Data Registry Voluntary Public Reporting Program: An Interim Report From the NCDR Public Reporting Advisory Group. J Am Coll Cardiol.

[3] Neil DA, Clarke S, Oakley JG (2004). Public reporting of individual surgeon performance information: United Kingdom developments and Australian issues. Med J Aust.

[4] Lancet. Public reporting of surgical outcomes. The Lancet. 2011;377(9772):1126. doi: 10.1016/S0140-6736(11)60446-7. 10.1016/S0140-6736(11)60446-7 21459198

[5] Hafner JM, Williams SC, Koss RG, Tschurtz BA, Schmaltz SP, Loeb JM (2011). The perceived impact of public reporting hospital performance data: interviews with hospital staff. Int J Qual Health Care.

[6] Sherman KL, Gordon EJ, Mahvi DM, Chung J, Bentrem DJ, Holl JL (2013). Surgeons’ perceptions of public reporting of hospital and individual surgeon quality. Med Care.

[7] Cacace M, Ettelt S, Brereton L, Pedersen J, Nolte E: How health systems make available information on service providers. Experience in seven countries. Santa Monica, CA: Rand Corporation.2011.http://www.rand.org/content/dam/rand/pubs/technical_reports/2011/RAND_TR887.pdf. Accessed 20 August 2016. 10.7249/rhq-01-01-11 PMC4945218 28083167

[8] James J. Health policy brief: public reporting on quality and costs. Health Aff. 2012;1–5.

[9] Shekelle P: Public performance reporting on quality information. In: Smith P, Mossialos E, Papanicolas I, Leatherman S, editors. Performance measurement for health system improvement. Cambridge: Cambridge University Press; 2009. p. 537–551.

[10] Ross JS, Williams L, Damush TM, Matthias M (2016). Physician and other healthcare personnel responses to hospital stroke quality of care performance feedback: a qualitative study. BMJ Qual Saf.

[11] Brekke KR, Nuscheler R, Straume OR. Gatekeeping in health care. J Health Econ. 2007;26(1):149–70. 10.1016/j.jhealeco.2006.04.004 16890313

[12] Binu VS, Mayya SS, Dhar M (2014). Some basic aspects of statistical methods and sample size determination in health science research. Int Q J Res Ayurveda.

[13] Narins CR, Dozier AM, Ling FS (2005). The influence of public reporting of outcome data on medical decision making by physicians. Arch Intern Med.

[14] Agency for Healthcare Research and Quality. Public Reporting as a Quality Improvement Strategy: A Systematic Review of the Multiple Pathways Public Reporting May Influence Quality of Health Care. 2011. http://www.effectivehealthcare.ahrq.gov/index.cfm/search-for-guides-reviews-and-reports/?productid=763&pageaction=displayproduct. Accessed 20 August 2016.

[15] Hibbard JH, Stockard J, Tusler M (2005). Hospital Performance Reports: Impact On Quality, Market Share, And Reputation. Health Aff.

[16] Marshall MN, Shekelle PG, Davies HTO, Smith PC (2003). Public Reporting On Quality In The United States And The United Kingdom. Health Aff.

[17] Rademakers J, Nijman J, Brabers AEM, de Jong JD, Hendriks M (2014). The relative effect of health literacy and patient activation on provider choice in the Netherlands. Health Policy.

[18] Wang X, Tang Y, Zhang X, Yin X, Du X, Zhang X (2014). Effect of Publicly Reporting Performance Data of Medicine Use on Injection Use: A Quasi-Experimental Study. PLoS One.

[19] Barr JK, Bernard SL, Sofaer S, Giannotti TE, Lenfestey NF, Miranda DJ (2008). Physicians’ Views on Public Reporting of Hospital Quality Data. Med Care Res Rev.

[20] Rechel B, McKee M, Haas M, Marchildon GP, Bousquet F, Blümel M (2016). Public reporting on quality, waiting times and patient experience in 11 high-income countries. Health Policy.

[21] Marjoua Y, Butler CA, Bozic KJ (2012). Public Reporting of Cost and Quality Information in Orthopaedics. Clin Orthop Relat Res®.

[22] Dehmer GJ, Drozda JP, Brindis RG, Masoudi FA, Rumsfeld JS, Slattery LE (2014). Public Reporting of Clinical Quality Data. J Am Coll Cardiol.

[23] Lamb GC, Smith MA, Weeks WB, Queram C (2013). Publicly Reported Quality-Of-Care Measures Influenced Wisconsin Physician Groups To Improve Performance. Health Aff.

[24] Smith MA, Wright A, Queram C, Lamb GC (2012). Public Reporting Helped Drive Quality Improvement In Outpatient Diabetes Care Among Wisconsin Physician Groups. Health Aff.

[25] Agency for Healthcare Research and Quality. Selecting Process Measures for Clinical Quality Measurement. 2016. https://www.qualitymeasures.ahrq.gov/help-and-about/quality-measure-tutorials/selecting-process-measures. Accessed 20 August 2016.

[26] Centers for Medicare & Medicaid Services. Process-Based Quality Improvement (PBQI) Manual. 2010.

[27] Yang L, Liu C, Wang L, Yin X, Zhang X (2014). Public reporting improves antibiotic prescribing for upper respiratory tract infections in primary care: a matched-pair cluster-randomized trial in China. Health Res Policy Syst.

[28] Kumpunen S, Trigg L, Rodrigues R. Public reporting in health and long-term care to facilitate provider choice. WHO Regional Office for Europe and European Observatory on Health Systems and Policies. 2014;1–48.

