# Supplementary material for: General practitioners’ perceptions of public reporting of institution and individual medicine prescribing data
Source: BMC Health Serv Res. 2016 Nov 9;16:641. doi: 10.1186/s12913-016-1893-5 (PMC5103380; doi:10.1186/s12913-016-1893-5)
Supplement: Additional file 1: — Appendix A: Questionnaire of General Practitioners’ Perceptions of Public Reporting. (DOCX 19 kb) [file 12913_2016_1893_MOESM1_ESM.docx]

**Appendix A:**

**Questionnaire of General Practitioners' Perceptions of Public Reporting**

Instructions: The following pages list some perceptions of public reporting medicine prescribing data. For each item, please read all choices, and then mark a “√” in the blank which best describes your feeling. This survey is anonymous and there's no right or wrong answer. Please do not skip any item.

| What do you think of public reporting medicine prescribing data: | not at all necessary | slightly necessary | somewhat necessary | very necessary | extremely necessary |
| --- | --- | --- | --- | --- | --- |
| 1 public reporting **institution-level** data is necessary |  |  |  |  |  |
| public reporting **individual-level** data is necessary |  |  |  |  |  |
| 2 third-party reporting **institution-level** data is necessary |  |  |  |  |  |
| third-party reporting **individual-level** data is necessary |  |  |  |  |  |
|  |  |  |  |  |  |
| When public reporting medicine prescribing data, do you concern about the following questions: | extremely concerned | moderately concerned | somewhat concerned | slightly concerned | not at all concerned |
| 3 patients can’t interpret or recognize **institution-level** data |  |  |  |  |  |
| patients can’t interpret or recognize **individual-level** data |  |  |  |  |  |
| 4 the **institution-level** data that public reporting is unreliable |  |  |  |  |  |
| the **individual-level** data that public reporting is unreliable |  |  |  |  |  |
| 5 a lack of appropriate **institution-level** medicine prescribe metrics |  |  |  |  |  |
| a lack of appropriate **individual-level** medicine prescribe metrics |  |  |  |  |  |
| 6 have no adequate risk adjustment when public reporting **institution-level** data |  |  |  |  |  |
| have no adequate risk adjustment when public reporting **individual-level** data |  |  |  |  |  |
| 7 have no adequate amount of prescriptions for statistical comparison at **institution-level** |  |  |  |  |  |
| have no adequate amount of prescriptions for statistical comparison at **individual-level** |  |  |  |  |  |
| 8 a lake of transparency of methodology when public reporting **institution-level** data |  |  |  |  |  |
| a lake of transparency of methodology when public reporting **individual-level** data |  |  |  |  |  |
|  |  |  |  |  |  |
| For public reporting medicine prescribing data, do you agree with the following opinions: | strongly disagree | disagree | neither agree nor disagree | agree | strongly agree |
| 9 Public reporting **institution-level** data can improve prescription quality |  |  |  |  |  |
| Public reporting **individual-level** data can improve prescription quality |  |  |  |  |  |
| 10 Public reporting **institution-level** data can promote GPs learn more knowledge about rational prescription of medicines |  |  |  |  |  |
| Public reporting **individual-level** data can promote GPs learn more knowledge about rational prescription of medicines |  |  |  |  |  |
| 11 Public reporting **institution-level** data can provide a sense of achievement to GPs |  |  |  |  |  |
| Public reporting **individual-level** data can provide a sense of achievement to GPs |  |  |  |  |  |
| 12 Public reporting **institution-level** data may lead to refuse high-risk patients |  |  |  |  |  |
| Public reporting **individual-level** data may lead to refuse high-risk patients |  |  |  |  |  |
| 13 Public reporting **institution-level** data may lead to improve numbers but not quality |  |  |  |  |  |
| Public reporting **individual-level** data may lead to improve numbers but not quality |  |  |  |  |  |
| 14 Public reporting **institution-level** data may penalize low performance GPs |  |  |  |  |  |
| Public reporting **individual-level** data may penalize low performance GPs |  |  |  |  |  |
| 15 Public reporting **institution-level** data may cause lose of volume of patients |  |  |  |  |  |
| Public reporting **individual-level** data may cause lose of volume of patients |  |  |  |  |  |
| 16 Public reporting **institution-level** data may increase medical disputes |  |  |  |  |  |
| Public reporting **individual-level** data may increase medical disputes |  |  |  |  |  |

17 When public reporting medicine prescribing data, what do you think are the important aspects? (Multi-select)

A. Internal review before public reporting

B. Third-party reporting

C. Educate patients on data interpretation

D. Present simplified data to patients

E. More comprehensive risk stratification and case-mix adjustment

F. Focus on process measures not outcome measures
